# Hedysari Extract Improves Regeneration after Peripheral Nerve Injury by Enhancing the Amplification Effect

**DOI:** 10.1371/journal.pone.0067921

**Published:** 2013-07-03

**Authors:** Zhiyong Wang, Peixun Zhang, Yuhui Kou, Xiaofeng Yin, Na Han, Baoguo Jiang

**Affiliations:** Department of Trauma and Orthopedics, Peking University People's Hospital, Beijing, China; University of Florida, United States of America

## Abstract

Radix Hedysari is an herbal preparation frequently used in traditional Chinese medicine. It can promote regeneration after peripheral nerve injury, but its effect on the amplification ratio (the ratio of distal to proximal fibers) during peripheral nerve regeneration has not yet been examined. In this study, we explored the effect of Hedysari extract on the amplification ratio in the peripheral nerve. Male Sprague-Dawley rats were separated into three groups at random: normal group (without surgery), model group (given sleeve nerve bridging surgery, but without adjuvant treatment) and treatment group (given sleeve nerve bridging surgery and then given Hedysari extract as adjuvant treatment). Twelve weeks after surgery, general observations, electrophysiological examination, histological analysis, morphometric measurements, and amplification ratio calculations were made. The results showed that nerve conduction velocity, the fiber and axon diameter, the g-ratio, the number of regenerating nerve fibers and the amplification ratio were better in the treatment group than in the model group, suggesting that Hedysari extract can effectively promote the growth of lateral buds in the proximal nerve stump and substantially improve the amplification effect during peripheral nerve regeneration.

## Introduction

Traditional Chinese Medicine has been used in China for hundreds of years and plays an important role in the clinical setting. Radix Hedysari is the root of Hedysarum polybotrys Hand.-Mazz., and has tonifying, diuretic and circulatory effects. It is blended into many Chinese medical formulations. Although the exact active ingredients are not very clear (only some Hedysari polysaccharides (HPS) and mineral elements have been roughly detected from Hedysari extract [1]), it was found that Radix Hedysari has a major effect on the circulatory, urinary and immune systems. Experimental studies have shown that Hedysari polysaccharides (HPS) can effectively promote peripheral nerve regeneration after nerve clamping injury, and can significantly improve the recovery of nerve function [2].

Peripheral nerve injury is quite common clinically. Traumatic injury, congenital anomalies and tumor extirpation may result in damage to or the complete sacrifice of critical nerves. The nerve stumps undergo Wallerian degeneration, which involves Schwann cell proliferation and a series of complex reactions after peripheral nerve transection. Proximal axons grow lateral buds toward the distal stump as a result of the growth promoting effects of various neurotrophic factors. The total number of lateral buds that proximal fibers grow is significantly more than the number of distal endoneurial tubes [3]–[4]. Numerous studies have demonstrated that using a few proximal fibers to bridge the distal nerve can increase the ratio of distal to proximal fibers (i.e., it has an *amplification effect*) during nerve regeneration, with a maximum amplification ratio of about 3.3. The amplified nerve fibers can improve the recovery of nerve structure and function. However, the number of regenerated nerve fibers has not yet been sufficient for clinical functional recovery. This requires us to identify a better method to promote the amplification effect. Previous studies on the nerve amplification effect focused on nerve growth postoperatively without providing adjuvant therapy. Consequently, we sought to determine whether the use of traditional Chinese medicine as an adjuvant treatment after surgery could encourage proximal axons to grow more lateral buds and whether it could enhance the amplification effect. In this study, we investigated the effect of Hedysari extract on the amplification phenomenon, and found that Hedysari extract can effectively improve peripheral nerve regeneration by enhancing the amplification effect.

## Materials and Methods

### Ethics Statement

The experimental procedures were carried out in accordance with the Chinese guidelines for the care and use of laboratory animals. The use of the animals was approved by the ethics committee and Experimental Animal Center of Peking University People's Hospital. All animal protocols were approved by the ethics committee of Peking University People's Hospital (Permit Number: 2011–16).

### Drug Preparation

Radix Hedysari, produced in Gansu province, China, was purchased from Min County in Gansu Province. Hedysari extract was made according to a traditional decocting method [1], [5], [6], as follows. 2 kg of dried Hedysari was boiled with 10 volumes of distilled water for 1 h and this procedure was carried out twice, and the mixed solution was concentrated to 1 g/ml (equivalent to the dry weight of Radix Hedysari) and stored at 4°C until use.

### Animals

Male Sprague-Dawley rats weighing 200–250 g were purchased from Beijing Vital River, and these animals were housed and cared for under specific pathogen-free laboratory conditions with free access to pellet food and water and kept under a 12 hours light/dark cycle. The rats were separated into three groups at random (six animals in each group). Every effort was made to minimize animal suffering and reduce the number of animals used, according to the Chinese guidelines for the care and use of laboratory animals. All animal protocols were approved by the ethics committee of Peking University People's Hospital.

### Materials

The materials used were: chitin biological absorbable tubes (ether-free chitin biological tubes; length, 8 mm; wall thickness, 0.2 mm; inner diameter, 1.5 mm); a Synergy electrophysiological instrument; a Leica tissue embedding machine; a Leica dissecting microscope; and a Leica image collection and analysis system.

### Surgical Procedures

**Figure 1:**
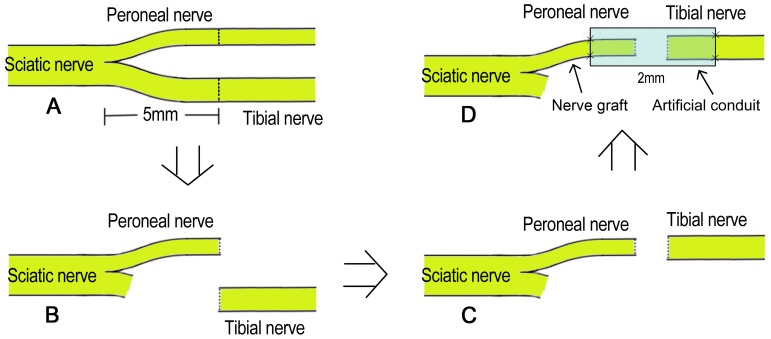
Surgical procedures. (A: The right sciatic nerve and its two main branches were exposed, then the common peroneal nerve and the tibial nerve were transected at 5 mm distal to the bifurcation; B: the proximal stump of the tibial nerve and the distal stump of the common peroneal nerve were ligated and stitched to the adjacent muscle; C: the proximal stump of the common peroneal nerve and the distal stump of the tibial nerve were aligned with each other; D: the proximal stump of the common peroneal nerve served as the donor nerve and was bridged to the distal stump of the tibial nerve with an artificial conduit).

Surgical procedures were permitted and approved by ethics committee and Experimental Animal Center of Peking University People's Hospital. Surgical procedures were performed in a specific pathogen-free animal laboratory using microsurgical techniques. Rats in the model and treatment groups were anesthetized with sodium pentobarbital (30 mg/kg, i.p.). Following anesthesia, the right limbs were sterilized and the sciatic nerve and its two main branches (the common peroneal nerve and the tibial nerve) were exposed. The common peroneal nerve and the tibial nerve were transected at 5 mm distal to the bifurcation. The proximal stump of the tibial nerve and the distal stump of the common peroneal nerve were ligated with 10–0 nylon sutures and stitched to the adjacent muscle. The proximal stump of the common peroneal nerve served as the donor nerve and was fixed to the distal stump of the tibial nerve. For this, we used biodegradable chitin conduits to create an artificial nerve graft; the conduits consisted of a polysaccharide shell exhibiting satisfactory biocompatibility and degradation characteristics. 10–0 nylon microsutures were used. The gap between the two nerve segments was kept at 2 mm. Subsequently, the muscle incision was sutured, and the wound was closed using 4–0 nylon sutures (Fig. 1). The untransected tibial nerves served as the normal group. Rats were then placed back into the cages in which they were raised, and the nerves were removed for examination 12 weeks after operation.

### Treatment Method

On the next day after surgery, rats in the normal and model groups were treated with 2 ml 0.9% NaCl by oral gavage once daily, and each rat in the treatment group was treated daily with 2 ml Hedysari extract liquid (1 g/ml) in the same manner and at the same time. The duration of oral gavage was 12 weeks.

### General Observations

After surgical operation, the general health of the animals was regularly monitored, including the degree of wound healing, the activities of the operated limbs, ulcer formation and necrosis on feet caused by self-biting of toes.

### Walking Track Analysis

Walking track analysis was performed for all the animals at 12 weeks post-surgery. Animals were allowed conditioning trials in a confined walking track (10×60 cm) darkened at one end. White paper that had the appropriate dimensions was placed on the bottom of the track. The rat’s hind limbs were dipped into black ink before the animal was placed at the entrance of the walking track. Foot prints appeared immediately on the paper when the rat walked down the track. Prints for measurement were chosen at the time of walking, based on clarity and completeness at a point when the rat was walking briskly. Animals in the normal group underwent the same procedure.

**Figure 2:**
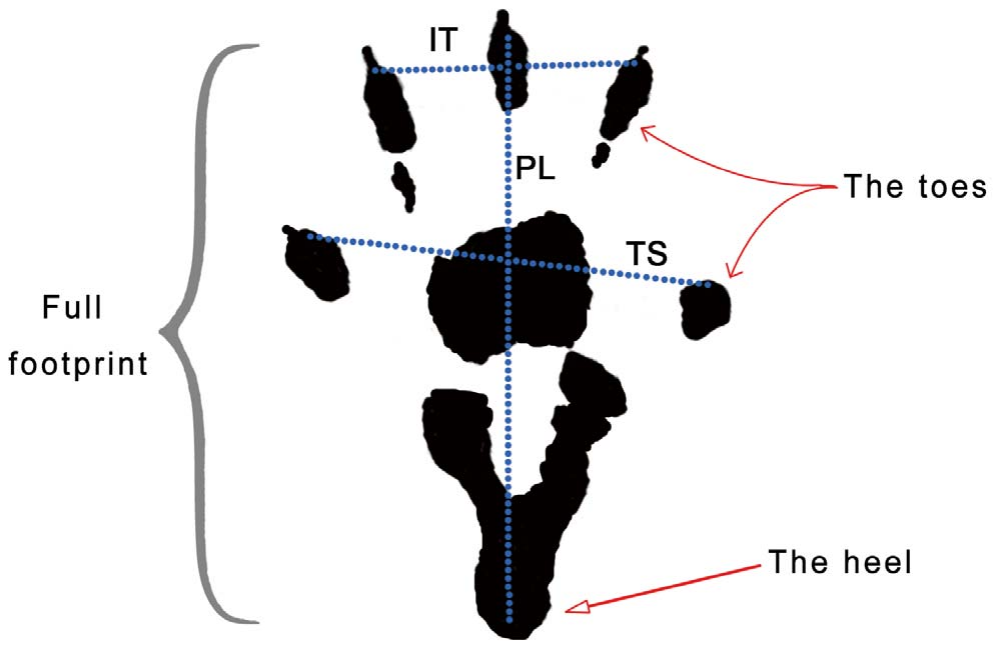
Walking track analysis. (The full right footprint. PL: print length; TS: toe spread; IT: intermediary toe spread).

Paired footprint parameters for print length (distance from heel to toe, PL), toe spread (distance from first to fifth toe, TS), and intermediary toe spread (distance from second to fourth toe, IT) were recorded for the left normal control foot (NPL, NTS, NIT) and the corresponding right experimental foot (EPL, ETS, EIT) for each rat. (Fig. 2).

Tibial function index (TFI) was calculated according to the Bain-Mackinnon-Hunter formula:

TFI = −37.2 ([EPL-NPL]/NPL) +104.4 ([ETS-NTS]/NTS) +45.6 ([EIT-NIT]/NIT) - 8.8.

### Electrophysiological Examination

**Figure 3:**
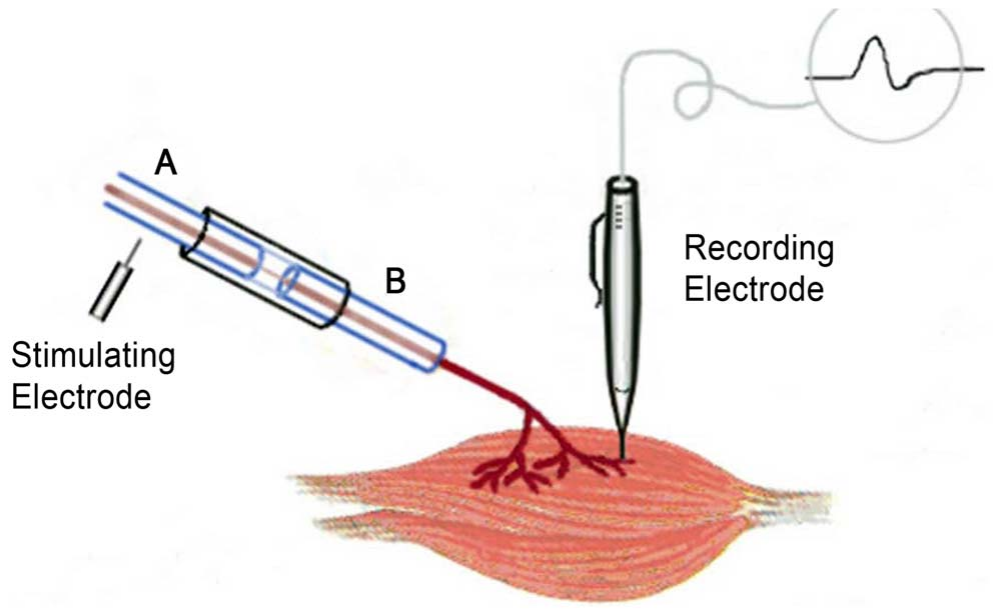
Electrophysiological test. (The stimulating electrodes were placed in A point and B point respectively, The recording electrode was placed in the gastrocnemius muscle. After stimulating A point and B point respectively, the conduction time was recorded automatically, then the distance between A point and B point was measured and the nerve conduction velocity was obtained.).

Electrophysiological examination was conducted 12 weeks post-surgery prior to sacrifice of the animals. The repaired tibial nerve was exposed, and stimulating electrodes were placed proximal and distal to the repair site in each group (Fig. 3, A point and B point). The recording electrode was placed in the gastrocnemius muscle, while the ground electrode was placed in the subcutaneous tissue between the stimulating and recording electrodes. Rectangular pulses (0.1 ms duration, 0.9 mA, 10 Hz, 6 continuous stimuli) were used. Upon stimulation of the repaired tibial nerves, the nerve conduction velocity (m/s) was obtained by dividing the distance between the two stimulating sites by the difference in the conduction time.

### Histological Analysis

**Figure 4:**
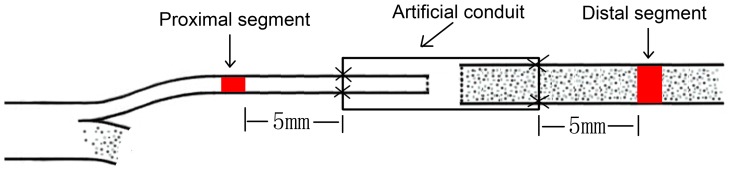
The excised specimen. (The proximal segment and distal segment were cut to be stained in osmium tetroxide.).

After electrophysiological examination, the entire nerve was removed from each rat. Tissues were then harvested and fixed in 4% paraformaldehyde in 0.1 M phosphate buffer for 24 h at 4°C. The nerves were then rinsed twice in water for 12 h. Two nerve segments were cut, one 5 mm proximal and one 5 mm distal to the chitin conduit (Fig. 4, red segments). Immediately after this step, each segment was stained in 1% osmium tetroxide for 12 h, then dehydrated through a graded series of ethanols, and then immersed in xylene, embedded in paraffin, and sliced into 5-µm cross-sections. Five slices were randomly selected from each nerve segment for observation. Images were acquired under a microscope, from which the total number and distribution of myelinated axons, fiber and axon diameter, g-ratio, and myelin thickness were evaluated.

We performed morphometric measurements using ImageJ software. The shortest lengths of the outer and inner margins of the myelin sheath were measured to determine the fiber diameter and axon diameter. After obtaining the fiber and axon diameter, myelin thickness and the g-ratio were calculated according to the above two indicators.

### Statistical Analysis

One-way analysis of variance was employed to compare the TFI, the motor nerve conduction velocity (MNCV), the fiber diameter, axon diameter and myelin thickness in each group. T-test was employed to compare the amplification ratio in the two surgical groups. A probability of *P*<0.05 was considered significant for all statistical comparisons. All values are presented as the mean ± SD.

## Results

### General Observations

**Figure 5:**
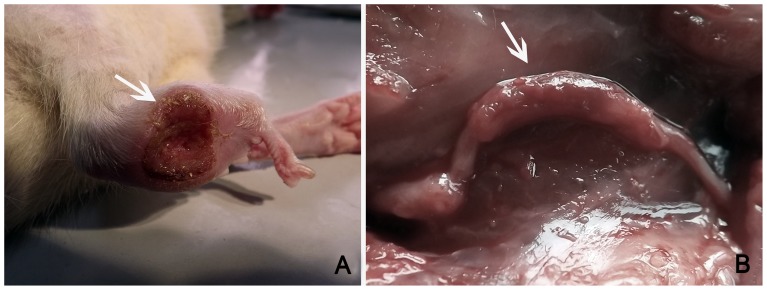
General characteristics. (A: Ulcer that has not yet healed; B: general observations 12 weeks after surgery).

Animals in the model and treatment groups were found to have postoperative lameness in the operated limbs, characterized by sagging ankles and awkward movements. Three rats exhibited the toe self-biting behavior, and their limbs appeared swollen and ulcerated 1 week after operation (1 animal in the model group and 2 in the treatment group), and 3 weeks later, the number of rats with ulcers increased. The motor function of the operated limbs began to gradually recover 4 weeks after surgical operation, and most ulcers gradually disappeared and eventually healed. However, at the end of the 12^th^ week post-surgery, the ulcers in three rats had still not healed (2 in the model group and 1 in the treatment group) (Fig. 5A). Twelve weeks after operation, the conduits had been absorbed partly, but retained their outline in the model and treatment groups, and there was a rich local blood supply at the site of the conduit in both surgical groups. The proximal peroneal nerve and distal tibial nerve were well connected by the conduit and in a good state of growth (Fig. 5B).

### TFI

**Figure 6:**
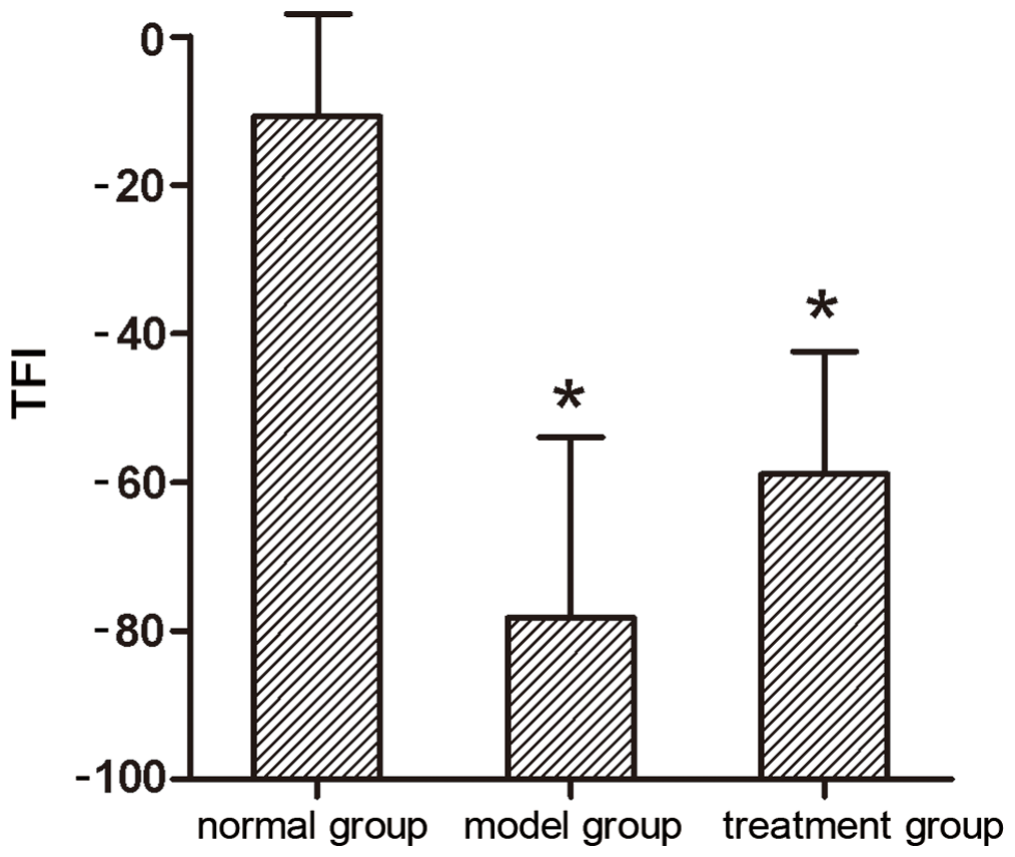
Tibial function index (TFI). (*P<0.05 versus normal group; ^▴^ *P*<0.05 versus model group).

Walking track analysis of rats that did not have ulcers and toe self-biting showed that TFI in the normal group, model group, and treatment group was −10.77±13.01, −78.26±24.31, and −58.90±16.45, respectively. The TFI in the normal group was significantly higher compared with the model group and the treatment group (P<0.05). The values in the model group and the treatment group had no significantly difference (P>0.05) (Fig. 6).

### MNCV

**Figure 7:**
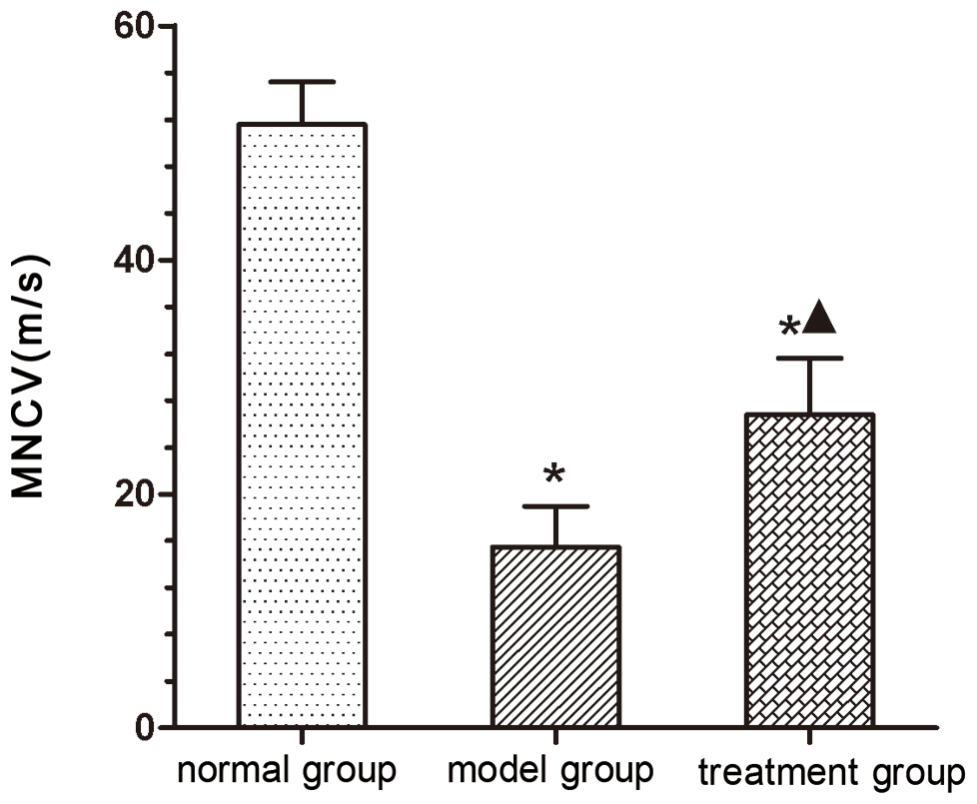
Motor nerve conduction velocity (MNCV). (*P<0.05 versus normal group; ^▴^ *P*<0.05 versus model group).

Electrophysiological assessment was conducted prior to sacrificing the animals 12 weeks post-surgery. MNCV in the normal group was 51.54±3.66 m/s; the value in the model group was 15.47±3.48 m/s; and the value in the treatment group was 26.75±4.84 m/s. MNCVs in the normal and treatment groups were significantly better than in the model group (*P*<0.05). The value in the normal group was significantly higher than in the treatment group (*P*<0.05) (Fig. 7).

### Osmium Tetroxide Staining

**Figure 8:**
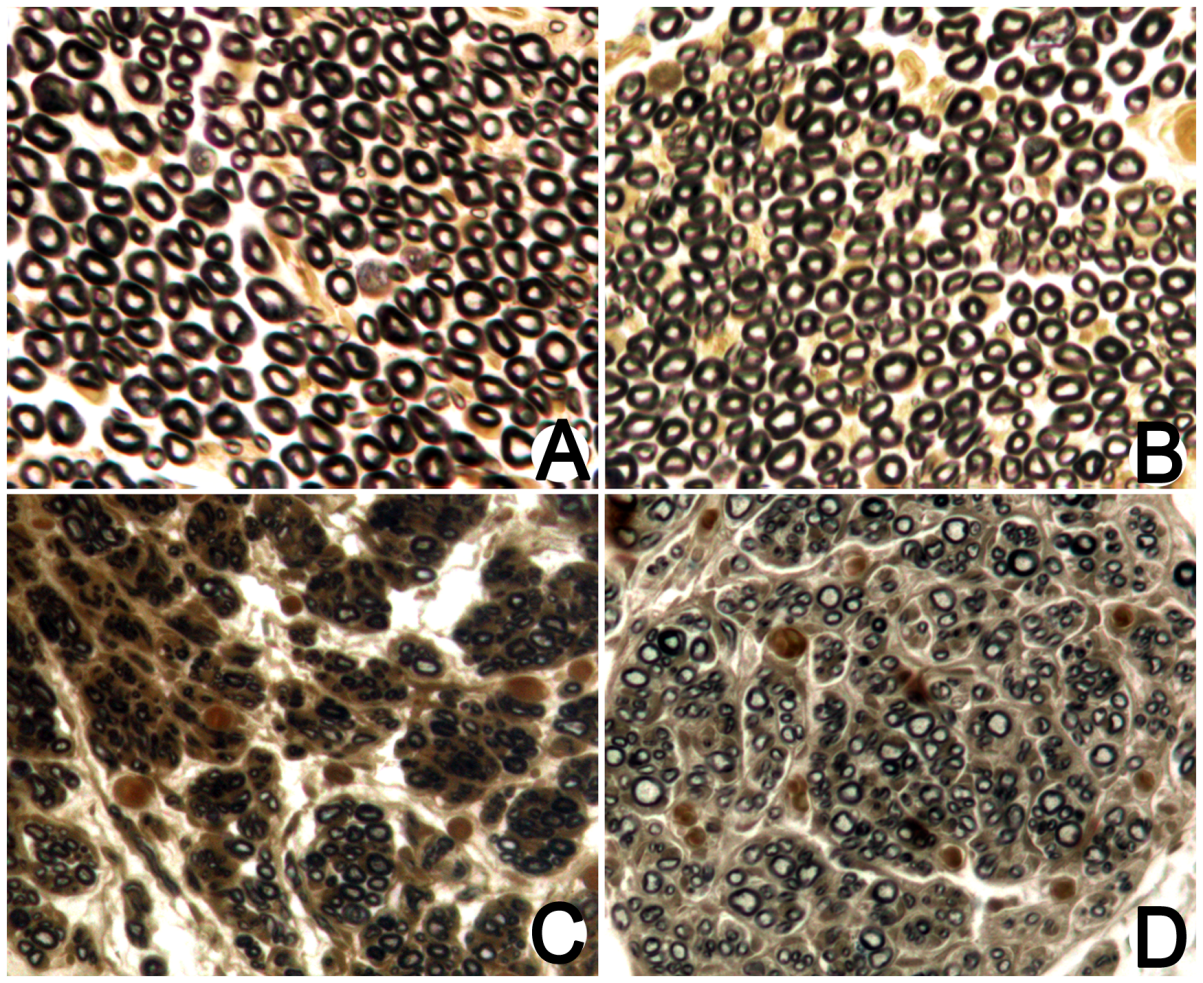
Osmium tetroxide staining in each group. (A: Proximal nerve segment; B: distal segment in the normal group; C: distal segment in the model group; D: distal segment in the treatment group).

Twelve weeks after surgery, the sectioned nerves from each group were stained with osmium tetroxide. In the proximal nerve segments in the normal group (Fig. 8A), good myelin regeneration with a uniform distribution was observed, and osmium tetroxide staining showed that the diameters and thicknesses of the myelin sheaths were uniform and regular. In the distal segments in the normal group (Fig. 8B), the characteristics were similar to those of the proximal segments. The characteristics of the proximal segments in the model and treatment groups were also similar to those in the normal group. In the distal segments in the model group (Fig. 8C), myelin regeneration was poor, with an uneven distribution and a low density. The myelin sheathes were highly variable, and osmium tetroxide staining showed that their diameters and thicknesses were uneven and generally smaller than those in normal tissue. In addition, the myelin sheathes were irregularly shaped and some myelin degeneration and necrosis could be seen. In the distal segments in the treatment group (Fig. 8D), myelin regeneration was better than in the distal segment of model group but poorer than in the normal group. The myelin distribution was not very uniform, but their density was higher than those in the distal segment of the model group. The diameters and thicknesses of the myelin sheaths were generally smaller than in normal tissue, but the diameters were larger than in the model group. A low level of myelin degeneration and necrosis was observed.

Distal nerve segments, which were 5 mm distal to the chitin conduits, were measured and analyzed in animals from each group. As shown in Table 1, fiber diameter in the normal group and treatment group was significantly larger than that in the model group (P<0.05), and the value in the treatment group was significantly lower than that in the normal group (P<0.05). Axon diameter in the normal group and treatment group was significantly larger than that in the model group (P<0.05), and the value in the treatment group was significantly lower compared with the normal group (P<0.05). G-ratio in the treatment group was significantly higher compared with the normal group and model group (P<0.05), and the values in the normal group and model group were not significantly different (P>0.05). Myelin thickness in the model group and the treatment group was significantly lower than that in the normal group (P<0.05), the values in the model group and treatment group were not significantly different (P>0.05).

**Table 1:** Morphometric measurements in all groups 12 weeks after surgery.

Group	Number ofslices pernerve segment	Number ofaxons quantifiedper slice	Fiber diameter(µm)	Axon diameter(µm)	G-ratio (axon diameter/fiber diameter)	Myelin thickness (µm)
Normal group	5	100	8.15±1.69	3.65±0.86	0.45±0.09	2.30±0.62
Model group	5	100	3.70±0.78*	1.80±0.60*	0.48±0.13	0.98±0.25*
Treatment group	5	100	5.30±0.85* ▴	3.00±0.64* ▴	0.57±0.08* ▴	1.15±0.28*

* *P*<0.05 versus normal group;

▴ *P*<0.05 versus model group.

### Amplification Ratio of Nerve Regeneration

The amplification ratio of nerve regeneration in the model group was significantly less than in the treatment group (*P*<0.05) (Table 2).

**Table 2:** Number of myelinated fibers and amplification ratio in each group.

Group	Number of nerves	Number of slices per segment	Number of proximal fibers	Number of distal fibers	Amplification ratio
Normal group	6	5	4984.27±294.07	5060.25±466.30	––
Model group	6	5	2016.94±181.38	2954.83±334.08	1.46±0.18
Treatment group	6	5	1979.01±199.63	3829.16±445.94	1.94±0.17*

Amplification ratio = number of distal fibers/number of proximal fibers. *P<0.05 versus model group.

## Discussion

Radix Hedysari is a crude medicine that has been frequently used by Chinese physicians for many centuries. It is a root of Hedysarum polybotrys HAND.-MAZZ., H. tanguticum Fedtsch or H. limprichtii Ulbr., which belong to the Leguminosae. It was reported that Radix Hedysari could act as a growth factor for B cells, increasing the number of total B cells as well as the proportion of activated B cells [7]. It also has a significant anti-CB4V effect at the cellular level [8], and it can prevent osteoporosis and disorders of bone metabolism caused by prednisone acetate [9]. In particular, Radix Hedysari plays an active role in the regeneration process after peripheral nerve injury. At present, it is known that Radix Hedysari can promote the proliferation of Schwann cells through the “receptor - cyclic adenosine monophosphate (cAMP) - protein kinase A (PKA) signaling pathway”, and that it can promote the differentiation of Schwann cells via the receptor tyrosine kinase (RTK) signaling pathway [10]. Schwann cells constitute the main component of the myelin sheaths during regeneration. As reported in another study, Radix Hedysari promotes nerve regeneration by promoting the secretion of neurotrophic factors, such as of Basic fibroblast growth factor (bFGF), Nerve Growth Factor (NGF) and Tropomyosin receptor kinase (Trk) [11], and these neurotrophic factors play positive roles in peripheral nerve regeneration. HPS, the main active ingredients in Radix Hedysari, have a protective effect on the inner cell membrane barrier [12]. HPS can improve the cytotoxicity of lymphokine activated killer cells (LAK cells) and peripheral blood mononuclear cells (PBMCs) [13], and also reduce free radicals by enhancing the activity of SOD and glutathione peroxidase [14]. These effects suggest that the mechanism by which Radix Hedysari promotes nerve regeneration is multifaceted, because traditional Chinese medicines are multi-component preparations that exert numerous effects. Radix Hedysari may stimulate changes in the microenvironment that promote nerve regeneration.

Several studies have demonstrated that Radix Hedysari can promote regeneration after peripheral nerve injury. It can effectively improve the motor nerve function index, nerve conduction velocity, and the number of regenerated myelinated nerve fibers [2], indicating that it has potential clinical therapeutic value for peripheral nerve injury. The regeneration of damaged nerves is a multifactorial process involving a variety of mechanisms, cells and factors [15]–[18]. Any treatment method using a single factor for adjuvant therapy is obviously one-sided, and may not be effective in targeting all pathological mechanisms involved in peripheral nerve injury. Recent studies have shown that locally applied neurotrophins can enhance the survival of damaged neurons and induce regrowth of lesioned axons in the central and peripheral nervous systems in rats [19]. However, the beneficial effect has been limited. Local application of neurotrophins after peripheral nerve sleeve bridging operation has been reported by several authors. Srinivas Madduri [20] advocated the inclusion of neurotrophic factors in the nerve conduit wall. Sun H. [21] implanted microspheres containing NGF into the neural tube before bridging the transected peripheral nerve, but found that there was no significant difference compared with the control group 3 months later with respect to nerve conduction velocity, muscle tension or muscle wet weight. Therefore, local applications that do not treat the body as a whole do not achieve the desired therapeutic efficacy. Traditional Chinese medicine may induce various biological changes, such as increasing the expression of neurotrophic factors, to provide a microenvironment that is conducive to nerve regeneration. Therefore, in this study, we chose Hedysari extract as an adjunctive treatment in the peripheral nerve amplification model, and the results show that Hedysari extract promotes the amplification effect in the regenerating peripheral nerve.

Previous studies have reported an amplification effect during nerve regeneration. In the initial stages of nerve regeneration after injury, one of the proximal nerve fibers grow several lateral buds, and these lateral buds grow into the distal myelin sheath tube [22]. According to this phenomenon, a relatively fine nerve can be used as a donor to repair the distal injured nerve in clinical situations. The donor nerve can grow more lateral buds than the host fibers, and these extend to the distal stump, thus ensuring the structural and functional recovery of damaged nerves at a lower cost. This present study confirms that there is an amplification effect during nerve regeneration.

The amplification effect during nerve regeneration is a complex and unique phenomenon. Distal nerves undergo Wallerian degeneration after peripheral nerve injury [23]. In this process, distal axons and myelin degenerate and collapse, and Schwann cells proliferate and form Bungner tubes in the original endoneurium to provide growth channels for the regenerating axons. Meanwhile, Schwann cells secrete a variety of neurotrophic factors and adhesion molecules that induce and promote newborn lateral buds to grow into the distal endoneurium tube [24]. Each axon in a proximal stump can grow several buds at the Ranvier node close to the stump [4], [25]. Generally during the regeneration period, when the lateral buds are blocked by scar tissue, connective tissue or other types of obstructions, or when they grow in the wrong direction, they cannot grow into the distal endoneurial tubes and they gradually degenerate. Therefore, when axons establish terminal structures, the number of collaterals is reduced to the usual single trunk [26]. However, when the number of distal endoneurial tubes is greater than that of donor axons (that is to say, the number of distal fibers is more than that of donor fibers), there is sufficient space for regenerating axons to grow into, and thus the nerve fiber amplification effect arises. In this study, the small peroneal nerve was treated as a donor nerve to connect to the distal tibial nerve via a chitin absorbable tube, and the results verified the nerve regeneration amplification effect.

An essential condition to ensure the amplification effect is the small gap sleeve bridging method, which is superior to the traditional epineurial or perineurial suture. The sleeve bridging method provides an appropriate room for nerve repair and regeneration, allowing the nerve to fully undergo selection and amplification in the regeneration compartment [27], [28]. Accordingly, in this study, chitin absorbable tubes were used, and the gap between the two nerve segments was kept at 2 mm (an appropriate gap for nerve regeneration and amplification).

This amplification effect may provide an opportunity for the development of a new method for the clinical repair of nerve damage, thus enabling the repair of nerve defects at a lower cost. This amplification effect can be achieved by promoting nerve regeneration using a small gap biomaterial bridge that encourages nerve growth. However, this approach does not yet effectively promote structural or functional recovery. We therefore hypothesized that an adjuvant therapy (e.g. Hedysari extract) given after surgical operation may induce lateral bud growth by proximal axons and stimulate the amplification effect, with the aim of promoting structural and functional recovery.

In the present study, our general observations revealed that two animals in the model group and one animal in the treatment group had unhealed ulcers within 12 weeks postoperatively. We speculated that Hedysari extract may exhibit neurotrophic effects and stimulate immune function to promote wound healing. However, an increased sample size is required to confirm this therapeutic effect because of the very limited number of rats with ulcers in this experiment. The TFIs in the model group and the treatment group had no significantly difference, but the value in the treatment group was higher than that in the model group (P = 0.194). Because rats (without ulcers and toe self-biting) which could leave complete footprints were few in number, an increased sample size is required for subsequent experiments to further confirm the functional recovery effects of Hedysari extract. Statistical analysis revealed that the MNCV in the treatment group was significantly higher than in the model group (*P*<0.05), and the nerve regeneration amplification ratio in the treatment group was significantly higher than in the model group (*P*<0.05). The fiber diameter, axon diameter and g-ratio in the treatment group were larger than those in the model group (P<0.05). These experimental results confirm the positive role of Hedysari extract in the amplification effect of peripheral nerves, thereby providing support for its use in the clinical repair of peripheral nerve injury. Our experiment demonstrates the efficacy of traditional Chinese medicine when all components, rather than individual constituents, in the medicinal preparation are used. Although HPS are able to protect the inner cell membrane barrier [12], enhance the cytotoxicity of LAK cells and PBMCs [13], and increase motor nerve function index, conduction velocity and the number of regenerated myelinated nerve fibers [2], the therapeutic effects are speculated to be inferior to Hedysari extract because HPS are only a limited number of ingredients in Hedysari extract. In contrast to HPS, Hedysari extract may contains all the active ingredients of Radix Hedysari (including HPS), better reflecting the overall therapeutic potential of traditional Chinese medicine.

Radix Hedysari has been used in animal studies on regeneration following peripheral nerve injury for many years, and it has been shown to effectively promote regeneration after peripheral nerve injury. Modified Formula Radix Hedysari (MFRH, including Radix Hedysari, Epimedium and Lumbricus) can be prepared as an extract according to a traditional decocting method that effectively promotes nerve regeneration. Some of the ingredients of Hedysari extract, HPS, can also effectively promote peripheral nerve regeneration. Therefore, based on these study results, we believe that Hedysari extract effectively and reproducibly promotes the regeneration of peripheral nerves.

In a study by Jiang and colleagues [29], the tibial nerve was transected and proximal donor nerves of varying diameters were attached to the distal tibial nerve. These authors observed that decreasing ratios of proximal donor nerve axon number to distal nerve axon number were associated with decreasing tibial nerve function. When the number of proximal donor nerve fibers was half the number of distal nerve fibers, the best structural and functional recovery was observed. Another conclusion from that study was that the maximum amplification ratio was 3.3. Our study results demonstrating that Hedysari extract can effectively promote the amplification effect should be explored further in subsequent experiments. For example, when Hedysari extract is used as adjuvant therapy, will the amplification ratio maximum exceed 3.3? Furthermore, is structure and function best recovered if fewer donor nerve fibers (less than half of the distal fiber number) are used to repair the tibial nerve?

In the present study, the concentration of Hedysari extract used was 1 g/ml. In subsequent experiments, higher concentrations of this traditional Chinese medicine will be used to further explore its effects and to determine the optimal dosage for the greatest peripheral nerve amplification effect. In addition, we investigated the effect of Hedysari extract at one time point only (12 weeks post-surgery) in this study. Further studies are therefore required to evaluate its long-term effects.

Like other traditional Chinese medicines, Hedysari extract can be prepared as a solution, sustained-release preparations, controlled-release preparations, powders, capsules and tablets. We believe that different preparations of the extract may have different levels of activity; for example, the activities of sustained-release preparations may be very different from solutions and powders. Exploring the appropriate preparations to be used in nerve repair will be important clinically.

More importantly, the specific active ingredients of Hedysari extract should be investigated in future studies because they remain undefined. Furthermore, the quantities of these active ingredients that reach the local sites of the damaged nerve should be estimated at different times. Further studies should also investigate the exact effect of Hedysari extract on Schwann cell proliferation and differentiation, and the key molecules in the nerve regeneration process. Therefore, the therapeutic potential of Hedysari extract for peripheral nerve injury is worthy of further investigation.

### Conclusions

Hedysari extract, a multi-component preparation that exert numerous effects, can enhance nerve amplification effect by encouraging proximal axons to grow more lateral buds, thus promote peripheral nerve repair at a lower cost.
